# Phosphoproteomics reveals essential regulatory roles of phosphorylation in marine oligotrophic bacteria

**DOI:** 10.1007/s42995-025-00305-w

**Published:** 2025-07-15

**Authors:** Yu Zhang, Yao-Hui He, Zhang-Xian Xie, Zhuo-An Bai, Guo-Sheng Hu, Ming-Hua Wang, Stephen J. Giovannoni, Da-Zhi Wang

**Affiliations:** 1 State Key Laboratory of Marine Environmental Science/College of the Environment and Ecology, Xiamen University, Xiamen, 361005 China; 2 MOE Key Lab of Rare Pediatric Diseases, Hengyang Medical School, University of South China, Hengyang, 421001 China; 3 School of Resources and Environmental Sciences/Key Laboratory of Rural Environmental Remediation and Waste Recycling, Quanzhou Normal University, Quanzhou, 362000 China; 4 School of Pharmaceutical Sciences, Fujian Provincial Key Laboratory of Innovative Drug Target Research, Xiamen University, Xiamen, 361102 China; 5 Department of Microbiology, Oregon State University, Corvallis, OR 97331 USA

**Keywords:** Marine oligotroph, SAR11 bacteria, Regulatory mechanism, Phosphorylation, Phosphoproteomics

## Abstract

Oligotrophic bacteria with reduced genomes have relatively few transcriptional regulators and are thought to rely more than other bacteria on post-transcriptional regulation to respond to environmental stimuli. SAR11 bacteria are the most abundant group of heterotrophic bacteria in marine planktonic systems and are a model for understanding genome reduction in other free-living microorganisms. Here, we report a comprehensive, quantitative protein phosphorylation profile for SAR11 strain HTCC1062 grown under various environmentally relevant conditions, including light/dark cycles, temperature differences, and nutrient limitations, to investigate phosphorylation dynamics in this streamlined organism. Nearly half of proteins encoded by the genome were detected in phosphorylated forms under at least one condition. 1014 Ser/Thr/Tyr phosphorylation sites were observed in 1576 phosphopeptides from 555 phosphoproteins. Protein phosphorylation was concentrated in proteins for functions associated with nutrient acquisition and growth, such as ABC transporters, RNA polymerase, and ribosomal proteins. Prominent patterns in protein phosphorylation were detected across a range of culture conditions. In these cells, which previously have been shown to continuously express nearly their entire proteome, protein phosphorylation was more dynamic than protein abundance, supporting the hypothesis that post-transcriptional regulation by protein phosphorylation might play a large role in modulating protein activity. Our findings support a regulatory model characterized by minimal variation in protein expression but extensive protein phosphorylation. This model diverges from bacterial regulatory paradigms reliant on transcriptional control, and may be relevant to understanding other abundant heterotrophs with reduced genomes.

**Supplementary Information:**

The online version contains supplementary material available at 10.1007/s42995-025-00305-w.

## Introduction

Metabolic regulation in response to environmental stimuli is thought to be a vital response of heterotrophic marine bacteria to the natural patterns of ocean variability. Gaining insights into the regulatory responses and strategies applied by these bacteria is critical to understanding their physiology, contributions to geochemical fluxes, and interactions with other cells and the environment (Noell et al. 2023a; Pisithkul et al. 2015). The concepts of copiotrophy and oligotrophy are frequently used to describe the lifestyle strategies in the ocean. Copiotrophic bacteria are easily cultured, grow rapidly at high nutrient concentrations (Cottrell and Kirchman 2016), and much of our current knowledge of bacterial regulation is derived from their study. More diverse and abundant oligotrophic bacteria thrive in oceanic environments with very low-nutrient availability and evidence suggests distinctive regulation in some oligotrophic taxa (Cottrell and Kirchman 2016; Lauro et al. 2009; Noell et al. 2023a, 2023b).

Generally, marine oligotrophs are non-motile, slow-growing, small in size, and have reduced genomes. A reduction in the number of transcriptional regulation elements, such as sigma factors and two-component systems, has been reported in streamlined oligotrophs, such as SAR11, SAR92, SAR86 and *Prochlorococcus* (Giovannoni et al. 2005, 2014; Lambrecht et al. 2020; Noell et al. 2023a). Several lines of evidence show little transcriptional response and even decoupled protein and transcript abundance in marine oligotrophs (Smith et al. 2010, 2013, 2016; Waldbauer et al. 2012), suggesting global reduction of transcriptional regulation commonly used by most bacteria to adjust their metabolic performance as their environment changes. Intriguingly, both transcriptomic and proteomic studies show that marine oligotrophs constitutively express the vast majority of genes (Noell et al. 2023a; Smith et al. 2010, 2013). Therefore, it is hypothesized that oligotrophs instead rely primarily on alternative forms of regulation, such as post-transcriptional regulation based on non-coding RNAs (e.g., riboswitches, small RNAs or antisense RNAs), post-translational modifications (PTMs) of proteins (e.g., phosphorylation), and kinetic regulation (Noell et al. 2023a; Voigt et al. 2014). Riboswitches and kinetic regulation in oligotrophs have been documented (Meyer et al. 2009; Noell et al. 2023a, 2023b; Sun et al. 2016; Tripp et al. 2009), while post-translational regulation is underexplored, and identification of protein phosphorylation events is barely reported in oligotrophs. The presence of kinases in marine oligotrophs (Giovannoni et al. 2005) suggests that protein phosphorylation has a regulatory role, complicated by the role of phosphorus as a critical limiting nutrient.

SAR11 bacteria, which are often perceived as model oligotrophs, are the most abundant bacterial lineage worldwide and thrive especially in the oligotrophic ocean (Giovannoni 2017). SAR11 bacteria are estimated to consume 6%-37% of marine gross primary production, and thus play a critical role in marine carbon cycle (White et al. 2019). The genome of a cultivated representative of SAR11 clade, “*Candidatus* Pelagibacter ubique strain HTCC1062” (HTCC1062 throughout) reveals that this clade has evolved a streamlined genome that has relatively few annotated transcriptional regulators (Giovannoni et al. 2005). Evidence suggests that transcriptional regulatory mechanisms are relatively few in these cells, and reserved for major stressors, such as nitrogen and phosphorus limitation, possibly because transcriptional responses are insufficiently rapid to respond to the fleeting opportunities presented by a patchy environment (Noell et al. 2023a).

SAR11’s global success is reliant on environmental adaptions to fluctuating light, temperature, and nutrient regimes. We postulated that protein phosphorylation might play a role in SAR11 bacteria by allowing them to respond rapidly to environmental stimuli. To investigate this hypothesis, we applied a phosphoproteomic approach to HTCC1062 grown under six different growth conditions mimicking the key environmental variables: light, which can support photoheterotrophy, temperature shifts, reflecting fluctuations in the cold-water niche of this strain, and nutrient limitation, reflecting the episodic scarcity of organic carbon or sulfur nutrients that is common in oligotrophic oceans. The comprehensive phosphoprotein profiles of HTCC1062 obtained across a variety of environmental changes suggest that protein phosphorylation plays a major regulatory role in this organism, significantly advancing our understanding of the specialized evolution and ecology of streamlined cells.

## Materials and methods

### Experimental design and growth conditions

*‘Ca. Pelagibacter ubique’* strain HTCC1062 was revived from frozen stocks, and cultured in an artificial seawater medium for SAR11 (AMS1) amended with pyruvate (100 µmol/L, as carbon source), glycine (25 µmol/L), methionine (25 µmol/L, as sulfur source), and modified vitamin mixtures (1 µmol/L pantothenate, 1 nmol/L biotin, 1 nmol/L pyrroloquinoline quinone, 1 nmol/L 4-amino-5-hydroxymethyl-2-methylpyrimidine and 1 nmol/L B_12_) according to a previously established protocol (Carini et al. 2013). Culture stocks (here called “normal” cells) were maintained using the amended AMS1 medium under 24-h darkness at 16 °C. Growth conditions across all treatments remained consistent unless otherwise specified.

To mirror in situ challenges from common environmental variations, six growth conditions were tested (Fig. S1). Control group (DSta) cells were grown under the same conditions as stock culture, described above. A light treatment (LSta) with a 12-h light:12-h dark cycle was used to investigate light-altered metabolism in this photoheterotrophic organism. HTCC1062 represents a cold-water ecotype of SAR11 found at high latitudes (Giovannoni 2017), and thus two temperatures, 8 and 12 °C (for groups of T8 and T12, respectively), that are lower than its common cultivation temperature (16 °C) were tested to study its response to a range of temperatures commonly encountered in the Oregon coastal environment from which it was isolated. Carbon (C_limit) or sulfur (S_limit) limitation was used to mimic episodic scarcity of these nutrients in the ocean. The defined medium contained pyruvate or methionine as sole carbon or sulfur sources respectively (Carini et al. 2013). Although methionine contains carbon, atypically for common heterotrophs, SAR11 cells do not grow on this compound alone, apparently because they cannot efficiently re-route methionine carbon to central metabolic pathways. Therefore, pyruvate in the C_limit group and methionine in the S_limit group were excluded to induce nutrient limitation. To obtain enough material for proteomic and phosphoproteomic analysis in nutrient-limited treatments, normal cultivated cells at the late-exponential phase were washed and resuspended in carbon or sulfur limited media to continue the cultivation until harvested. The cells in all groups were harvested in the early stationary growth phase via centrifugation (13,500 g for 2 h). Cell densities were monitored with a CytoFLEX S flow cytometer (Beckman Coulter) after staining with SYBR green I as described (Carini et al. 2013). Cell samples were immediately frozen at − 80 °C until analysis.

### Sample preparation for proteomic and phosphopeptide enrichment analysis

Protein extraction was performed based on previous studies (Li et al. 2019). Briefly, crude proteins were extracted from the cell pellets with the reagent of TRIzol according to the manufacturer’s instruction, and precipitated in ice-cold acetone at − 20 °C overnight. Protein pellet was collected via centrifugation at 20,000 g for 20 min and resuspended in rehydration buffer (7 mol/L urea, 2 mol/L thiourea, 40 mmol/L Tris and 2% sodium dodecyl sulfate). Protein concentration was determined using the 2D Quant protein assay kit (GE Healthcare) according to the manufacturer’s instruction. After a 30 min reduction with 20 mmol/L dithiothreitol and another 30 min alkylation with 50 mmol/L iodoacetamide, a subsample of 2 mg protein from each sample was loaded onto a 10 kD centrifugal filter device (Millipore) for protein digestion based on the filter aided sample preparation method (Gao et al. 2018; Liu et al. 2021). In brief, the protein sample was sequentially washed with 4 mL of 8 mol/L urea for six times and 4 mL of 50 mmol/L ammonium bicarbonate twice. Enzymes of trypsin and Lys-C (Promega) in 1.5 mL of 50 mmol/L ammonium bicarbonate were added at a ratio of 1:50 (enzyme/protein) at 37 °C for 16 h to cleave proteins into peptides. Peptides were washed twice with 1 mL of 50 mmol/L ammonium bicarbonate from the filter to a new collection tube after spinning at 14,000*g*, desalted with Sep-pak C18 (Waters) and eluted with 70% acetonitrile (ACN). The eluted peptides were lyophilized and stored at − 80 °C. Except 150 µg peptides for the proteomic analysis, the remaining were used for phosphoproteomic analysis. The phosphopeptide enrichment was performed using High-Select Fe-NTA kit (Thermo Scientific) according to the manufacturer’s instruction.

## LC–MS/MS analysis

MS analysis was performed on a nanoU3000 UHPLC system in line with an Orbitrap Fusion Lumos equipped with a nanoelectrospray source (Thermo Fisher Scientific). The peptides were reconstituted in 0.1% of formic acid (FA) with 2% of ACN, loaded onto a C18 column (75 µm × 50 cm, 2 µm particles, Thermo Fisher Scientific) with buffer A (0.1% FA, 2% ACN), and separated at a flow rate of 300 nL/min in a linear gradient from 8 to 26% of buffer B (0.1% FA, 80% ACN) in 125 min followed by another linear increase to 42% of buffer B in 25 min. The eluted peptides were analyzed using Orbitrap Fusion Lumos in a data-dependent manner alternating between full-scan MS and MS/MS scans with a dynamic exclusion of 15 s. The temperature of the ion transfer capillary was 300 °C, and the spray voltage was 2.2 kV. Survey full-scan MS spectra (350–1500 m/z) were collected at a resolution of 120,000. Selected ions were sequentially fragmented at a normalized collision energy of 30% and a resolution of 30,000 under a mode of high-energy collision dissociation. Unassigned ions or those with a charge of 1 + or more than 7 + were excluded for MS/MS scan.

### Protein identification

An in-house version of Proteome Discoverer (version 2.4) was used to search the acquired MS/MS spectra against a custom non-redundant protein database combined 1,354 protein entries predicted from HTCC1062 genome deposited in the National Center for Biotechnology Information (NCBI) GenBank with the renewed sequences based on our unpublished proteogenomic study (Supplementary Table 1). The data search was performed in default parameters except for precursor mass tolerance of 20 ppm, fragment mass tolerance of 0.02 Da, trypsin and Lys-C as the enzymes with three allowed miss cleavages, oxidation (M) and N-terminal acetylation as variable modifications with an addition of phosphorylation (S, T, Y) during the phosphoproteomic analysis, and carbamidomethylation (C) as fixed modification. Peptides with at least six amino acids in length and a false discovery rate (FDR) of < 1% were applied to filter the identified peptides and proteins, and at least one unique peptide was considered as confident protein identification.

### Bioinformatic analysis

Protein annotations were derived through parallel integration of NCBInr, Gene Ontology (GO) and Kyoto Encyclopedia of Genes and Genomes (KEGG) databases. To resolve inconsistencies among databases, protein annotations were primarily relied on pathway-mapped to KEGG for pathway centric annotations, functional specified using GO terms, and BLASTP-aligned against NCBInr for baseline validation. Proteins with at least two unique peptides were considered for protein quantitation. Differentially expressed proteins were defined using a difference of 1.5-fold compared to the control group (DSta) and *p*-value < 0.05. The KEGG pathway enrichment analysis was conducted and evaluated based on hypergeometric test.

### Statistical analysis

Triplicate samples were performed for each growth conditions. Statistical analyses were conducted in R software (v4.3.1) using stats and rstatix packages. Statistical significance for group comparisons was determined using two-tailed Student’s *t* test for pairwise comparisons (i.e., LSta vs. DSta for light variation, C_limit vs. DSta for carbon limitation, and S_limit vs. DSta for sulfur limitation) or one-way ANOVA for multi-group analysis (i.e., T8, T12 and DSta groups for temperature gradients).

## Results

### Kinase and phosphatase in SAR11 bacteria

Only three kinases (PhoR, RegB and ChvG), predicted to act on histidine residues, and one kinase (RsbW) predicted to act on serine/threonine residues, were detected at both the gene and protein levels in HTCC1062 (Table 1), implying regulatory mechanisms based on phosphorylation might be simple in SAR11. None of the six phosphatases annotated in the genome is predicted to dephosphorylate protein substrates, suggesting that an undescribed protein dephosphorylates proteins in this strain.

**Table 1:** Known protein kinases in strain HTCC1062 found in the proteome

Gene name	type	Locus	Proteome	Function
rsbW	Ser/Thr kinase	SAR11_0057	Yes	Anti-sigma regulatory factor
phoR	His kinase	SAR11_1180	Yes	Phosphate regulon
regB	His kinase	SAR11_0447	Yes	Sensor histidine kinase
chvG	His kinase	SAR11_0198	Yes	Two-component sensor histidine kinase

### Extensive protein phosphorylation in SAR11 bacteria

**Fig. 1:**
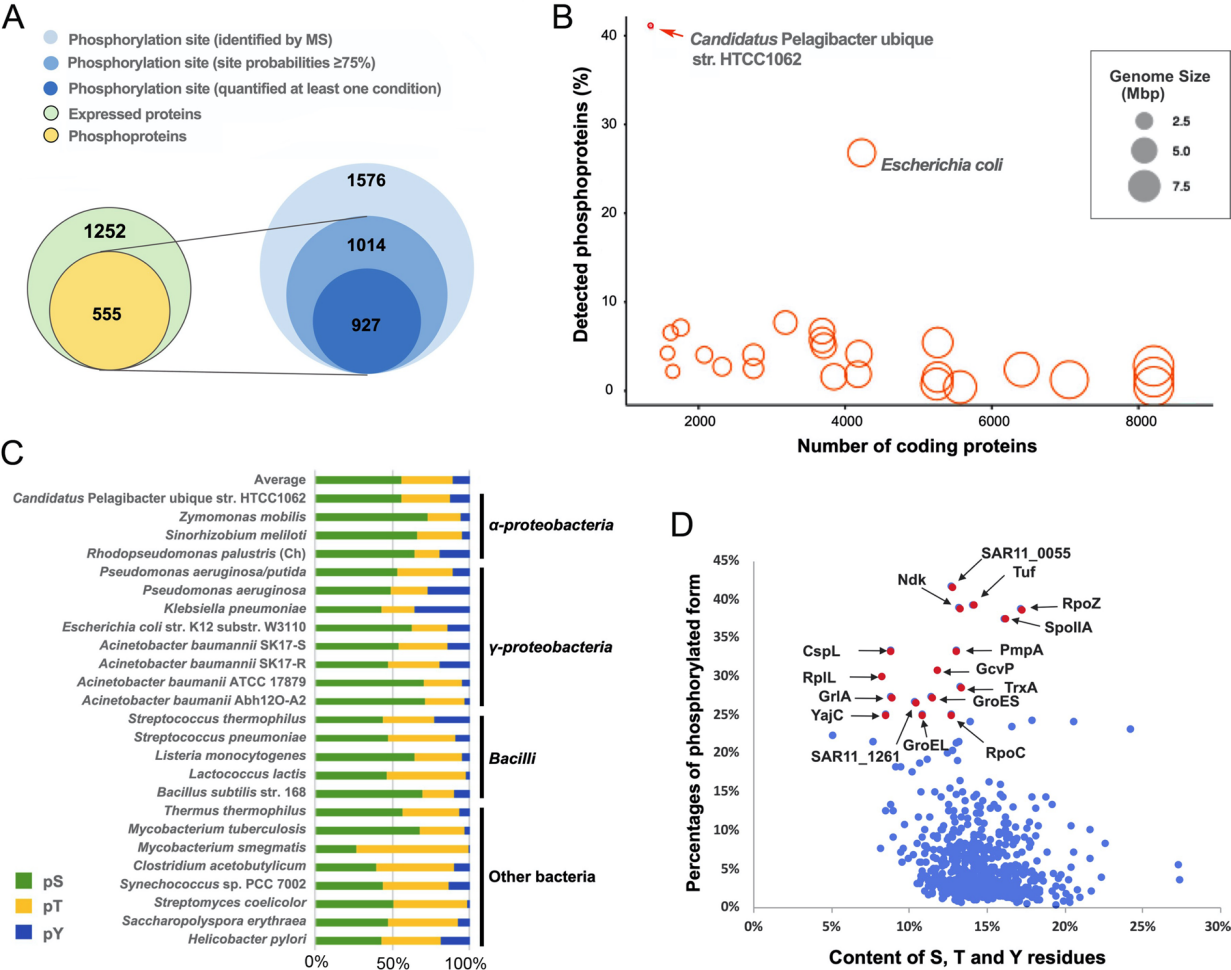
Characterization of the phosphoproteome of SAR11 strain HTCC1062. **A** Overview of proteome and phosphoproteome from the same protein materials of the SAR11 strain HTCC1062 growing in the six tested conditions. **B** Phosphoproteins of HTCC1062 ranks the top percentage of genome coding proteins among published bacterial phosphoproteomes. Each circle indicates one bacterial phosphoproteome, and ranks in the order of genome size from small to large. The red circle indicates the position of HTCC1062. **C** Distribution of phosphoserine (pS), phosphothreonine (pT) and phosphotyrosine (pY) in phosphoproteomes of HTCC1062 and other published bacteria. **D** Scatter plot indicates the relationship between content of three residues (S, T and Y) and percentage of phosphorylated form among S, T and Y residues in a protein. Red dots indicate proteins are highly phosphorylated on S, T or Y residues

To study the regulatory strategy of SAR11 bacteria, HTCC1062 cells grown under the six conditions mimicking key environmental variables of light, temperature and organic carbon and sulfur nutrients were harvested for proteome and phosphoproteome analyses with a label-free approach (Fig. S1). Proteomic analysis at a false discovery rate (FDR) of 1% resulted in identification of 1,252 proteins across all conditions (Supplementary Table 2, Fig. 1A), while a total of 1576 phosphopeptides corresponding to 1249 unique peptides were detected at an FDR of 1% (Supplementary Table 3, Fig. 1A). Among them, 1,014 phosphorylation sites of class I (unambiguously localized, single residue localization probability ≥ 75%) were determined in 555 phosphoproteins, of which 235 proteins contained multiple phosphorylation sites (Supplementary Table 4). We further identified 927 phosphorylation sites that were unambiguously localized on 519 proteins (Supplementary Table 5). Based on these data, we investigated patterns of PTM by phosphorylation in HTCC1062 to understand the role phosphorylation might play.

Although fewer phosphoproteins were observed in HTCC1062 than the extensively studied *Escherichia coli* (555 Vs. 1133) (Lin et al. 2018), the detected phosphoproteins in HTCC1062 accounted for a higher percentage of genome-predicted proteins than in *E. coli* (41% vs. 27%), easily ranking SAR11 above other bacteria in the proportional size of their phosphoproteome (Fig. 1B and Supplementary Table 6). We sampled a range of conditions and biological replicates to increase the detected number of phosphoproteins, but detected high numbers of phosphoproteins (425 ± 76) even in single phosphoproteome experiment. All detected phosphorylation sites were distributed on the residues of serine (pS, 56.1%), threonine (pT, 31.4%) and tyrosine (pY, 12.5%) (Fig. 1C), close to the average ratio of pS: pT: pY (54.0:32.9:10.5) in other bacterial species (Yague et al. 2019). Notably, most phosphoproteins contained a similar content of S/T/Y residues (10%-20%) but varied widely in the percentage of pS/pT/pY, some of which, such as Tuf, RplL RpoC, and GroEL, were highly phosphorylated at S/T/Y residues (Fig. 1D and Supplementary Table 7). Taken together, the results suggest that cellular functions are modulated by Ser/Thr/Tyr phosphorylation in SAR11 bacteria on a large scale.

**Fig. 2:**
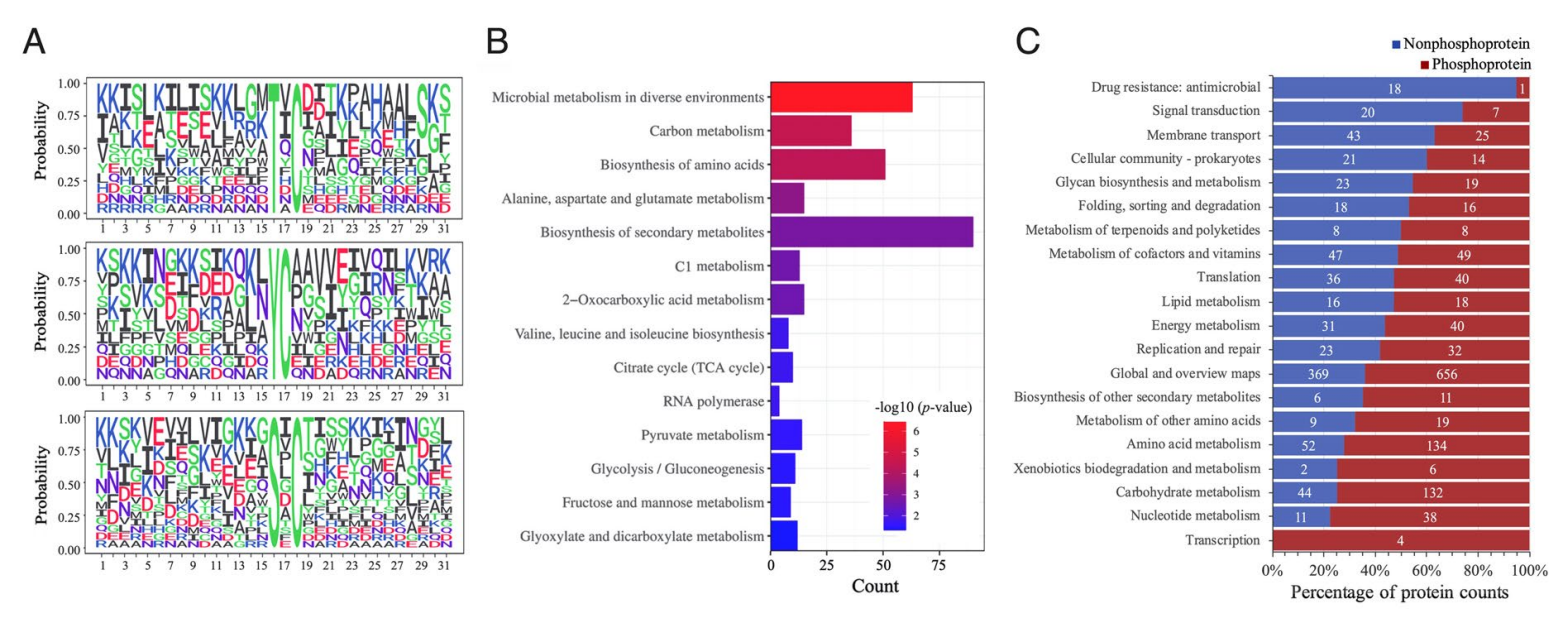
Phosphorylation motifs and functional distribution of phosphoproteins in HTCC1062. **A** Three examples of phosphorylation motifs detected in HTCC1062 phosphoproteomes using the Motif-X analysis. **B** KEGG enrichments of detected phosphoproteins with motif features. The color scale indicates different thresholds of the *p*-value with logarithmic transformation (− log10). **C** Distribution of detected phosphoproteins and nonphosphoproteins in each KEGG category. Numbers within the bars show counts of nonphosphoproteins (blue) and phosphoproteins (red)

To check whether phosphorylation site consensus sequences motifs were over-represented in phosphoproteins of SAR11 bacteria, motif structures were extracted with Motif-X. A total of 30 motifs were enriched, and 27 out of them associated with 422 phosphorylation sites contained a cysteine residue at + 1 or more positions near pS/pT/pY as in the three examples shown in Fig. 2A. A high frequency of cysteine flanking phosphosites was also reported in the *Synechococcus* sp. strain PCC 7002, although preferred motifs were not extracted in that study (Yang et al. 2013). The phosphorylation sites with motif structure were enriched in cellular functions, such as carbon metabolism, amino acid metabolism, biosynthesis of secondary metabolites, and transcription (RNA polymerase) (Fig. 2B). Consistently, high percentages of phosphoproteins were distributed in a variety of KEGG pathways, such as transcription, metabolism of amino acids, nucleotides, carbohydrates and lipids, translation, replication and repair, protein folding, membrane transport, and signal transduction (Fig. 2C and Supplementary Table 8).

**Fig. 3:**
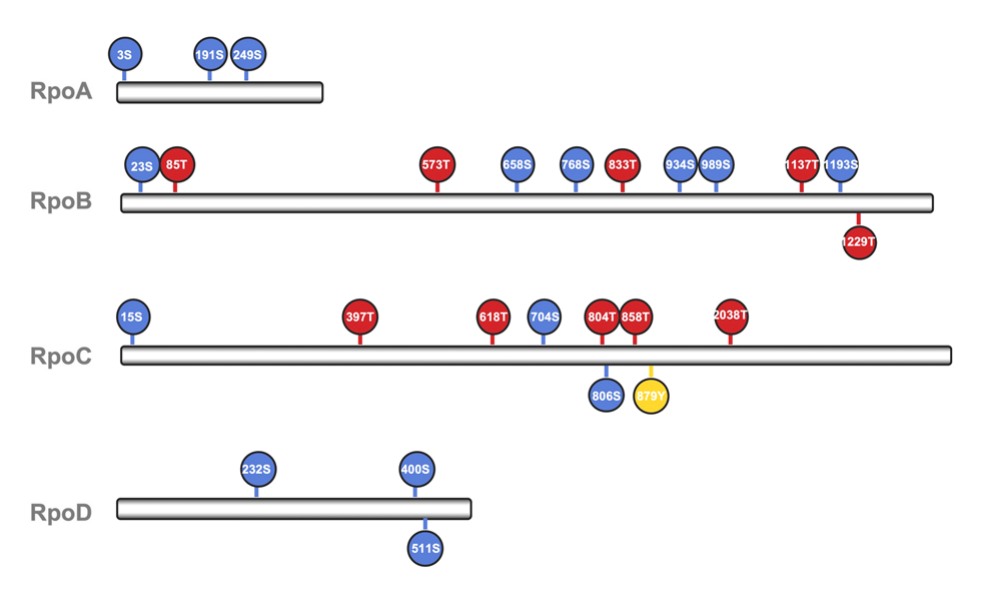
Phosphorylation of transcriptional machinery components detected in phosphoproteome of SAR11 strain HTCC1062. Multiple phosphorylation sites are identified in RNA polymerase core subunits RpoA, RpoB, RpoC, and the sigma factor RpoD. Phosphoserine (pS), phosphothreonine (pT) and phosphotyrosine (pY) are highlighted in blue, red and yellow circles, respectively. In each circle, the number in front of the letter indicates the position of the amino acid residues in a protein

**Fig. 4:**
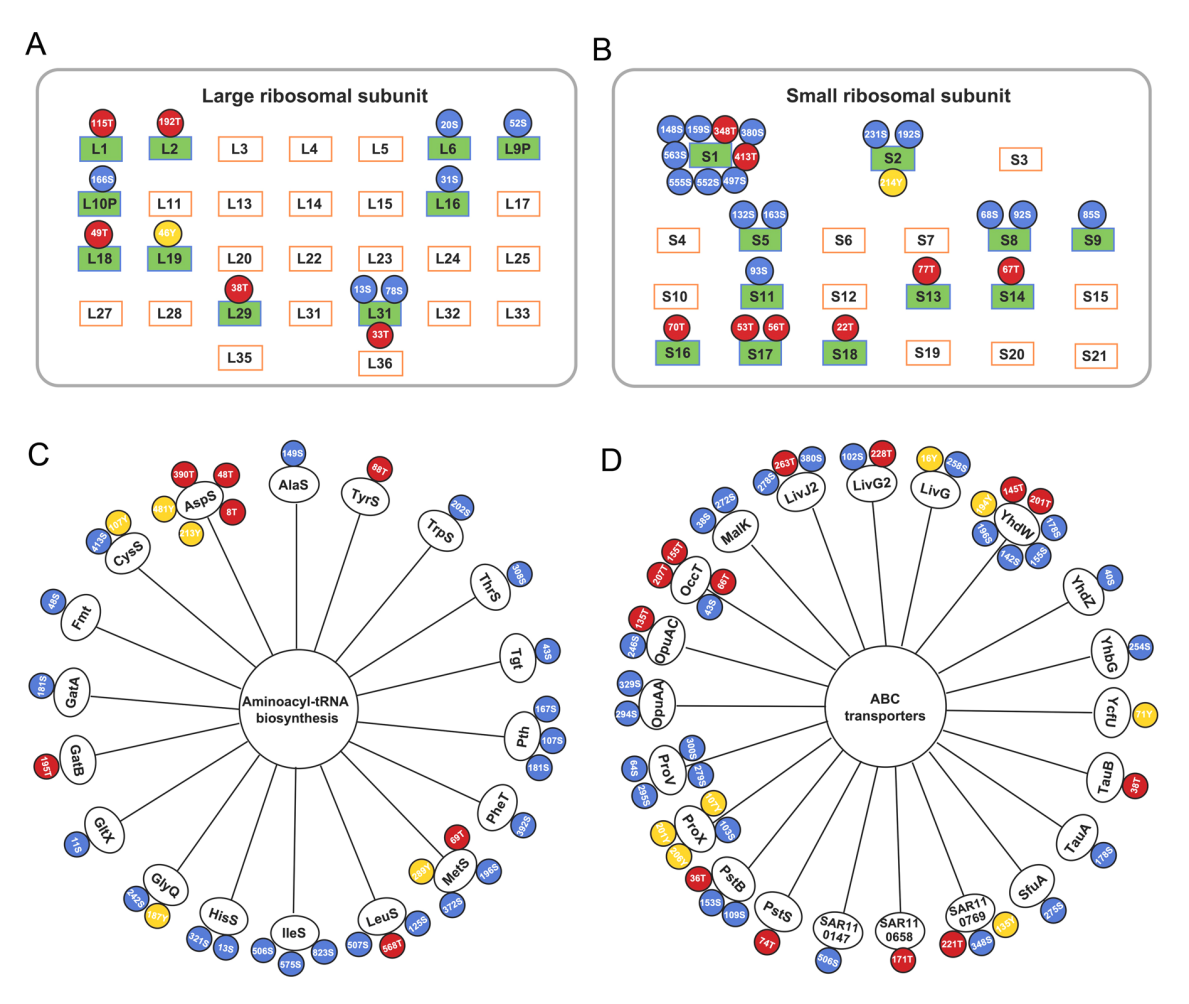
Detection of phosphoproteins relevant to translation and transport in phosphoproteome of SAR11 strain HTCC1062. Phosphorylation sites are detected on large (**A**) and small (**B**) ribosomal protein subunits, aminoacyl-tRNA biosynthesis-related proteins (**C**), and ABC transporters primarily functioning as ATP binding or periplasmic substrate-binding components (**D**). The color and position of each phosphoserine (pS), phosphothreonine (pT) and phosphotyrosine (pY) in a protein are highlighted in the same manner as in Fig. 3

We detected two phosphorylation sites in two response regulators NtrX and RegA, three in RpoD (σ^70^), and one in RpoH (σ^32^) (Fig. 3 and Supplementary Table 5). NtrX and RegA are, respectively, involved in regulating responses to nitrogen limitation and cellular redox, and sigma factors control the binding of RNA polymerase to gene promoters for transcriptional initiation. In addition to sigma factors, RpoA, RpoB, RpoC and RpoZ, all subunits of the key transcriptional enzyme RNA polymerase were phosphorylated at multiple sites (Fig. 3). Phosphorylation of bacterial RNA polymerase has been reported in RpoC in four bacteria (Yague et al. 2019). Phosphorylation of additional subunits and more phosphorylation sites in RpoC, as we report in this study, is a new finding that suggests transcription could be heavily phosphoregulated in SAR11 bacteria. Furthermore, frequent phosphorylation of ribosomal proteins (at least 21 out of 51) and aminoacyl-tRNA biosynthesis-related proteins (including 18 aminoacyl-tRNA ligases and synthetases), which are essential for ribosome structure and protein synthesis, was also detected (Fig. 4A, B, C). Moreover, a total of 21 ABC transporter proteins were phosphorylated, including mineral and ion transporters, sugar transporters, amino acid transporters, and lipoprotein transporters (Fig. 4D).

### Dynamic phosphoproteome under variable growth conditions

**Fig. 5:**
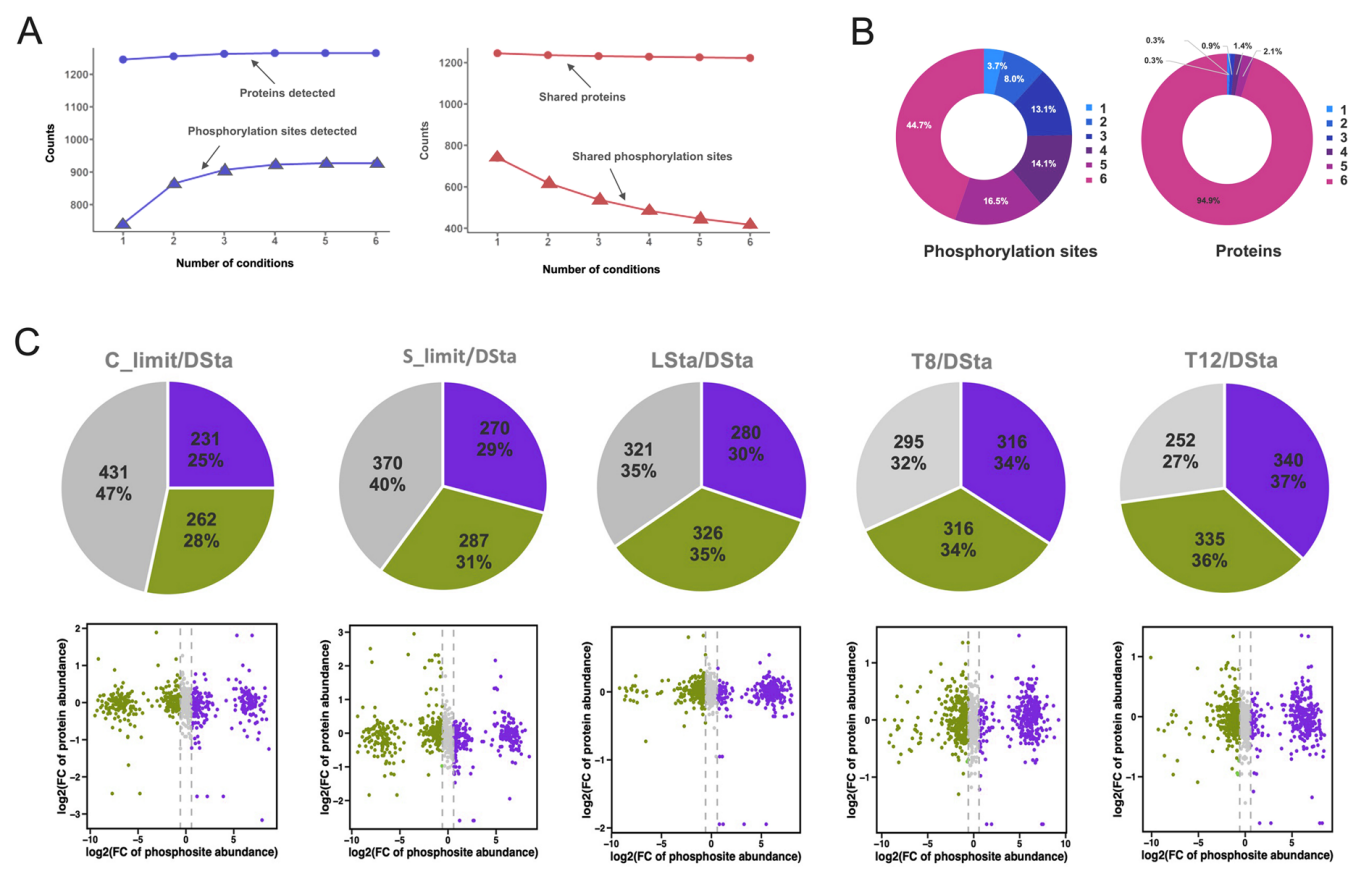
Dynamic changes of HTCC1062 phosphoproteomes. **A** An accumulative increase (left panel) in numbers of detected proteins (circle) or phosphorylation sites (triangle) while a decrease trend (right panel) in numbers of shared proteins or phosphorylation sites for each added culture condition. **B** Doughnut charts show count percentages of the phosphorylation sites (left) or proteins (right) shared among conditions. Category labeled “1” indicates unique phosphoproteins or proteins detected in one of the conditions while others with labels of 2–6 indicate phosphoproteins or proteins commonly detected among 2–6 conditions. **C** Pie plots show the distribution of up-regulated (purple), down-regulated (green) and insignificantly (gray) changed phosphorylation sites at a level of 1.5-fold change when compared with the DSta group. Numbers in the pies indicate the counts or percentages of phosphorylation sites in each category. Scatter plots show the abundance relationship between phosphorylation site and its protein expression

We quantified variation in protein phosphorylation and abundance across a range of growth conditions and found the numbers of detected proteins and phosphorylation sites increased with the number of conditions we tested and asymptotically tended to a maximum as four conditions were analyzed (Fig. 5A). In the quantitative proteome dataset, 1252 proteins, accounting for 95% of the proteins identified, were expressed in all six conditions, while only four proteins were detected only in one specific condition. In contrast, 419 phosphorylation sites, accounting for 45% of the phosphorylation sites, were phosphorylated in all growth conditions, whereas less than 4% were exclusively phosphorylated in a one condition (Fig. 5B). These results indicate that phosphoproteome of HTCC1062 is much more dynamic than proteome. Using the DSta (darkness and at stationary phase) group cells as a control, differentially expressed proteins and variable phosphorylation sites (fold change > 1.5 and *p* value < 0.05) were profiled in other growth conditions (Fig. 5C and Supplementary Table 9). Most phosphoproteins with differentially phosphorylated sites did not differ significantly in protein abundance, and fold changes of phosphorylation sites were commonly larger than their protein counterparts. Globally, across treatments, quantitative protein abundance profiles in SAR11 bacteria were remarkably similar while phosphorylation patterns were more variable, further confirming that the phosphoproteome was more dynamic than the proteome. In contrast, significant differences among treatments are observed in both proteomes and phosphoproteomes in the copiotrophic bacterium, *E. coli* (Lin et al. 2018). Although the comparative cell biology of bacteria is limited by the very small number of cell types that have been investigated, our findings align with other evidence of distinct regulatory differences between oligotrophs and copiotrophs (Noell et al. 2023a). Moreover, a scatter plot of phosphoprotein abundances and phosphorylation sites also displayed distinct differences in global patterns of phosphorylation between nutrient limitation, light, and temperature treatments. Collectively, our results suggest that SAR11 bacteria might significantly rely on dynamically changing patterns of Ser/Thr/Tyr phosphorylation to post-transcriptionally regulate cellular metabolic activity in response to environmental changes.

**Fig. 6:**
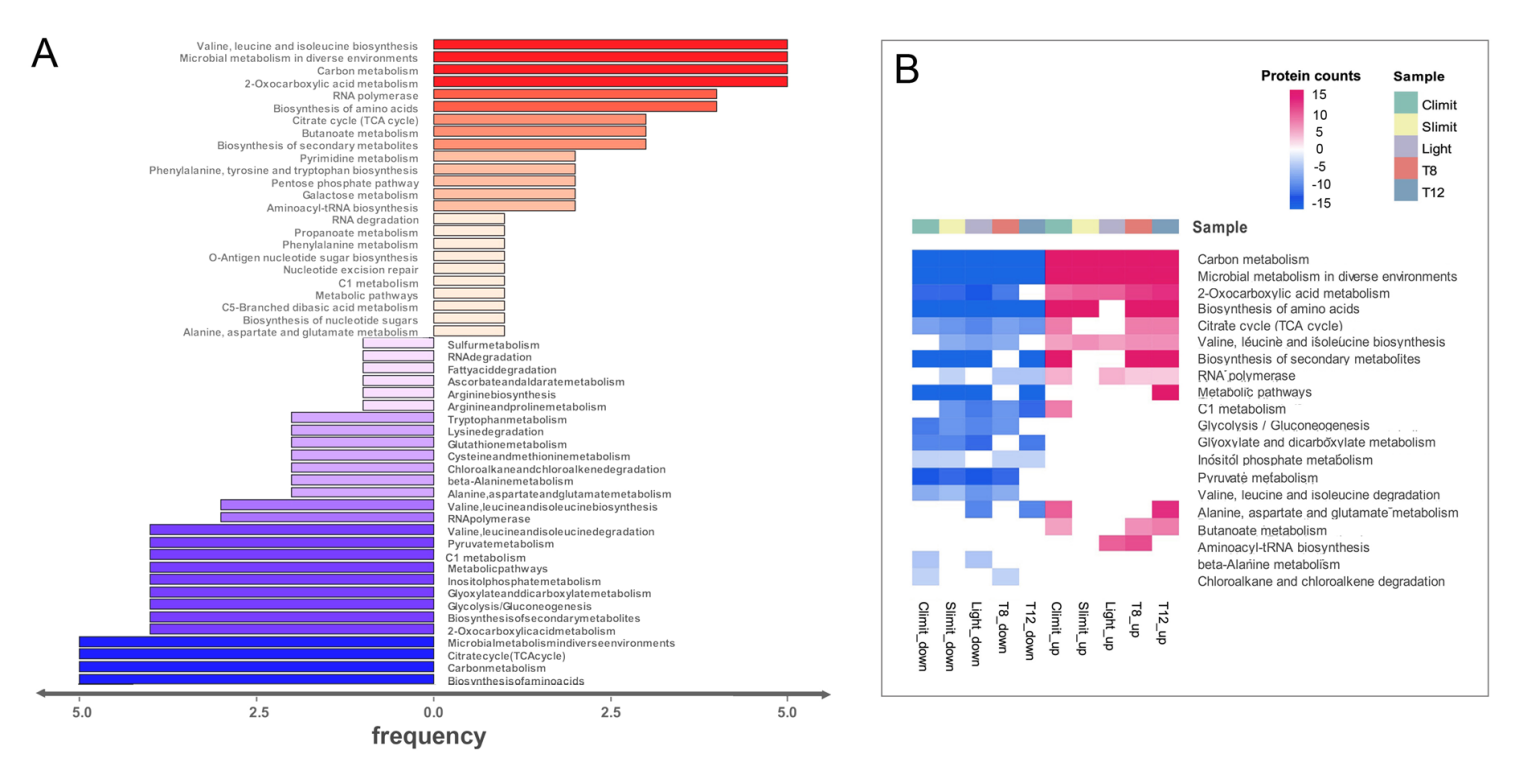
KEGG enrichment analysis of differential phosphorylation sites detected in phosphoproteome of SAR11 strain HTCC1062. **A** Overview distribution of differential phosphorylation sites enriched in KEGG pathways of which in red are enriched with up-regulated phosphorylation sites while blue with down-regulated ones. Frequency represents how many conditions the enriched pathway occurs. **B** Enrichment of KEGG pathway for up-regulated (in magenta) and down-regulated (in blue) phosphorylation sites present in each condition. Color gradient in the array indicates the count of phosphoproteins in an enriched pathway

**Fig. 7:**
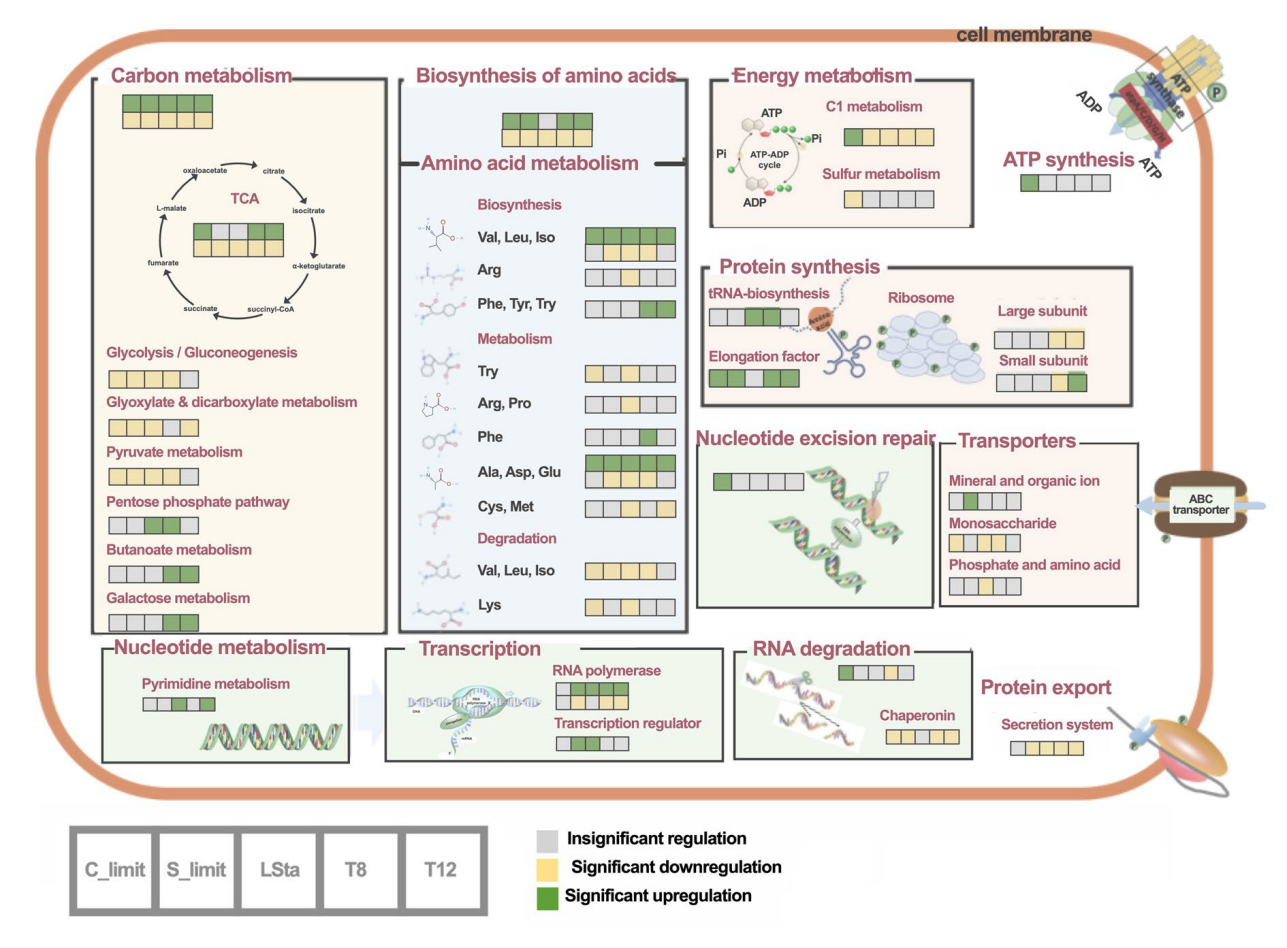
Overview of phosphorylation-regulated KEGG pathways in SAR11 strain HTCC1062 across growth conditions. Green squares indicate significant upregulation of phosphorylation sites (*p*-value < 0.05) in pathways relative to the DSta group, yellow squares represent significant downregulation, and gray squares denote insignificant difference

**Fig. 8:**
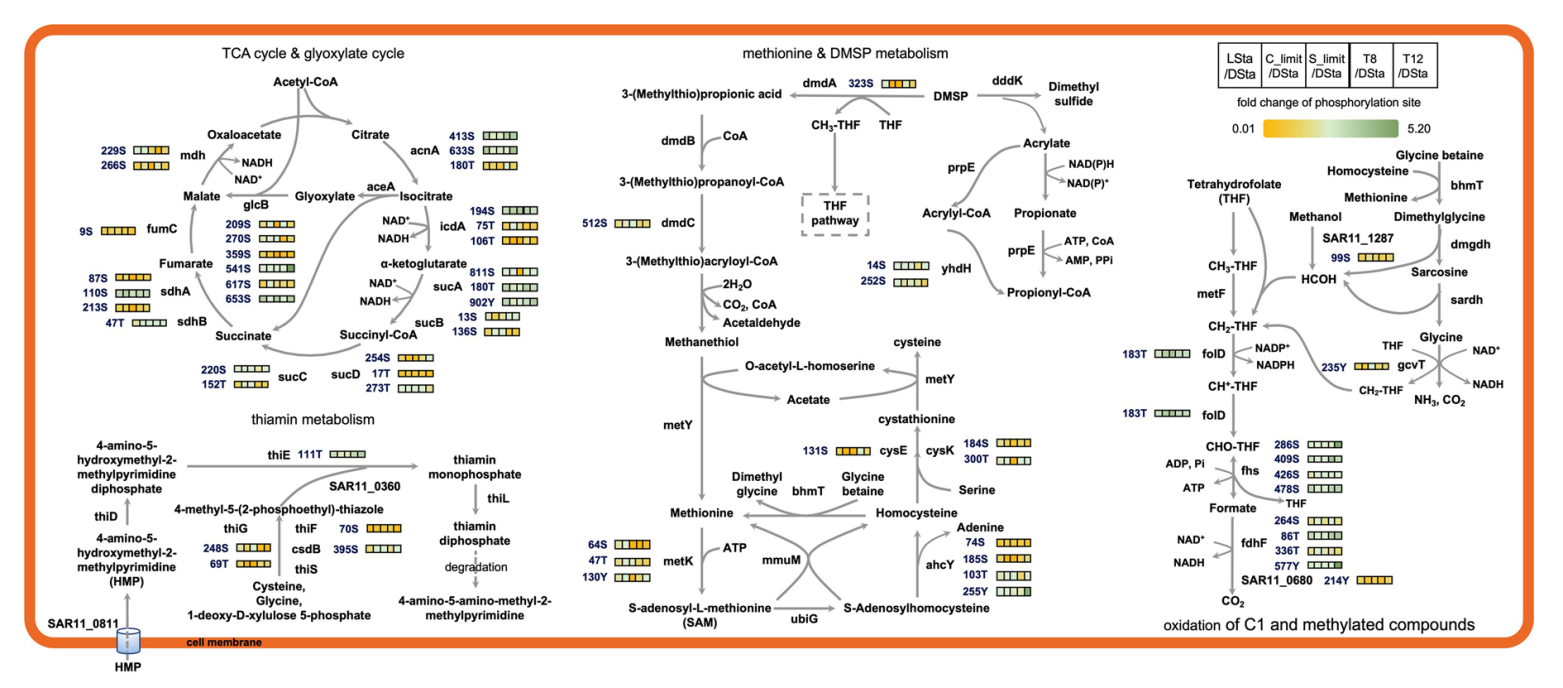
Schematic diagram of phosphorylation-modulated key metabolic pathways in SAR11 strain HTCC1062. Variation of a phosphorylation site on a specific residue of a protein across growth conditions is indicated using heatmap squares with a green-to-yellow color gradient. Representing upregulation (green) and downregulation (yellow) relative to the DSta group

Consistent with qualitative results, KEGG pathway enrichments for differential phosphorylation sites in both global (Fig. 6A**)** and condition-specific (Fig. 6B**)** analyses indicated phosphorylation mainly in carbon metabolism, amino acid metabolism, carbohydrate metabolism, transcription, translation and ABC transporters. Interestingly, both up- and down-regulated phosphorylation sites were simultaneously observed in central metabolism, for example in citrate cycle and amino acid metabolism (Fig. 7). The key pathways containing differential phosphorylation sites across conditions were summarized (Fig. 8). Phosphorylation of serine residue at positions of 209, 270, 359, 541, 617 and 653 significantly varied in the GlcB protein in the glyoxylate cycle. Differential phosphorylation of GcvT, FolD and Fhs was observed in the experiments, indicating phosphoregulation of folate biosynthesis. Multiple phosphorylation sites on formate dehydrogenase, a key enzyme in the C1 oxidation pathway, also were differentially phosphorylated. In the metabolic pathway of dimethylsulfoniopropionate (DMSP), significant variations of pS in DmdA at position 323, in DmdC at position 512, and in YhdH at positions of 14 and 252 were detected. Moreover, variation of phosphorylation sites was also present in proteins of AhcY, CysK, CysE, ThiEFG and CsdB that are involved in intermediate metabolites of cysteine and thiamin. Overall, dynamic phosphorylation sites in many key enzymes suggest that phosphorylation on serine, threonine or tyrosine residues plays an important role in energy production, carbon oxidation, and reduced sulfur and vitamin metabolisms (Fig. 8).

## Discussion

### Reduction of kinases and phosphatases in SAR11 inconsistent with its phosphoproteome

In general, protein phosphorylation and dephosphorylation are catalyzed by kinases and phosphatases, respectively (Macek et al. 2019). Many bacteria, for example cyanobacteria, contain many kinases with specificity for residues of serine, threonine and tyrosine (Yang et al. 2013), However, only one kinase (RsbW), predicted to act on serine/threonine residues, was detected in the HTCC1062 genome (Table 1). In contrast, the evidence suggests multiple regulons are controlled by protein phosphorylation, encompassing 1014 Ser/Thr/Tyr phosphorylation sites on 555 proteins. Histidine kinases are sensor proteins ubiquitously found in prokaryotes, where they function in two-component signal transduction pathways by autophosphorylating a key histidine residue. Three histidine kinases are present in the HTCC1062 genome but detection of phosphorylated histidine residues is incompatible with the acid environment in liquid chromatography tandem–mass spectrometry (LC–MS/MS) protocols, and therefore they were not measured in our experiments (Yague et al. 2019).

Bacterial serine/threonine/tyrosine kinases can phosphorylate a broad spectrum of substrates (Cousin et al. 2013). The RsbW kinase, often referred to as an anti-SigB factor, is a group of diverse serine/threonine kinases that are known to activate an antagonist of SigB, a general regulator of stress responses in many bacteria (Dufour and Haldenwang 1994). Interestingly, there is absence of known protein phosphatases in the HTCC1062 genome in contrast with the observed dynamic phosphorylation of proteins across conditions, suggesting that unknown mechanisms are involved in dephosphorylation. Of course, current approach of phosphatases is largely relied on conserved catalytic domain structures reported in literature or protein database, which could deny the discovery of atypical phosphatases whose structural features are beyond our current knowledge (Sadatomi et al. 2013). Another possibility is that the genome reduction of SAR11 may drive the evolution of multifunctional enzymes with uncharacterized phosphatase activity. A similar example of multifunctional protein has been reported in a SAR11 transporter (Noell and Giovannoni 2019). More efforts should be devoted to extensive exploration and substrate characterization of new protein phosphatases and kinases. Collectively, these results suggested that protein modification by phosphorylation, controlled by a small set of kinases, could be the feature of the streamlined cells of the SAR11 clade (Giovannoni et al. 2005).

### Phosphorylation modulated diverse metabolism in SAR11

Our findings suggest that protein phosphorylation is responsible for controlling vital cell functions, such as transcription, translation, carbon and amino acid assimilation, and responses to environmental stimuli such as macronutrient supply, light and changing temperature (Figs. 3, 4, 7, 8). Dynamic protein phosphorylation dependent on growth conditions has been reported in *E. coli* (Lim et al. 2015; Soares et al. 2013). In *E. coli* K12, protein phosphorylation is extensively involved in variety cellular functions, including central carbon metabolism and housekeeping processes, such as DNA metabolism, transcription, carbohydrate metabolism, and translation (Lin et al. 2015). Phosphoproteins have been identified in carbon and nitrogen metabolisms, and photosynthesis in cyanobacteria (Yang et al. 2013), and include ribosomal proteins, cell division proteins, RNA polymerases, enzymes from glycolysis, gluconeogenesis, elongation factors, and the citrate cycle in other bacteria (Yague et al. 2019). Thus, protein phosphorylation is known to play diverse, vital roles in many fundamental metabolic processes in bacteria.

SAR11 bacteria are known to have a streamlined genome with a reduced number of transcriptional regulation proteins, only two sigma factors (heat shock factor σ^32^ and a σ^70^) and four two-component regulatory systems (*PhoR*/*PhoB*, *NtrY*/*NtrX*, *envZ*/*OmpR* and *RegB*/*RegA*) (Giovannoni et al. 2005). The observations of phosphorylation events in σ^32^, σ^70^, NtrX and RegA suggest that transcriptional regulation, a process that requires several minutes to exert an effect, might be coupled with Ser/Thr/Tyr phosphorylation, a potentially much more rapid modulator of function, to adjust transcriptional activity in response to environmental perturbations. Ser/Thr/Tyr phosphorylation in two-component systems has also been reported in *Synechococcus* and *E. coli* (Lin et al. 2018; Yang et al. 2013). Our results suggest the possibility of coupling between global post-translational regulation by Ser/Thr/Tyr phosphorylation and two-component systems of regulation, which has not been considered previously.

Phosphorylation of ribosomal proteins, found in HTCC1062 in the present study (Fig. 4A, B), has been reported in several bacterial species, but the patterns of phosphorylation reported were distinctly different among the organisms tested (Soung et al. 2009; Yague et al. 2019). Phosphorylation of ribosomal proteins has been shown to alter ribosomal activity (Mikulik et al. 2011). We speculate that ribosomal protein phosphorylation could benefit these bacteria by reducing investments in ribosome turnover and instead controlling ribosome activity to modulate the rate of new protein production. Besides ribosomal proteins, the occurrence of phosphorylation events in RNA polymerase, aminoacyl-tRNA ligases and synthetases indicates that biosynthetic pathways leading to proteins in SAR11, from transcription to translation, are heavily phosphorylated, suggesting that phosphorylation plays a fundamental role in the control of SAR11 protein synthesis.

Differential phosphorylation of ABC transporters has also been observed in *E. coli*, suggesting that phosphorylation is used to regulate substrate uptake (Lin et al. 2018). In HTCC1062, YhdW, an amino acid transporter for amino acids, was phosphorylated at multiple sites (Fig. 4D). Uncoupling of HTCC1062 YhdW protein and transcripts has been reported (Smith et al. 2013), and it was postulated that short-term changes in the activity of the transporter that includes the YhdW protein might be attributable to PTM (Noell et al. 2023b). Our finding of YhdW phosphorylation supports this hypothesis. SAR11 bacteria are reported to abundantly express diverse ABC transporters in both laboratory and in situ environments (Sowell et al. 2009), and are specialized in utilizing labile dissolved organic matter (Giovannoni 2017). The phosphorylation of specific serine, threonine or tyrosine residues in almost all ABC transporters observed in this study suggests that phosphorylation is significantly involved in the regulation of organic carbon uptake and oxidation in SAR11 cells.

More importantly, the protein abundance profile of HTCC1062 cells was less dynamic than protein phosphorylation. One example is that most of enzymes in both the citrate and glyoxylate cycles changed insignificantly in abundance but were dynamically phosphorylated across the conditions we tested. The control of carbon flux through metabolism in SAR11 by regulation of the glyoxylate cycle appears to be a metabolic strategy for responding to nutrient deficiency (Beier et al. 2015). The multiple detections of differential phosphorylation sites in GlcB suggest post-translational control of metabolic flux from glyoxylate to malate, a route postulated to channel exogenous precursors of glycine biosynthesis to energy metabolism when intracellular supplies of glycine are adequate (Tripp et al. 2009). Similarly, we speculate that the observed variation in phosphorylation of enzymes of the citrate cycle might control the dynamics of intermediate metabolites, many of which serve dual functions in anaplerotic metabolism and carbon oxidation for energy. Another example is DMSP metabolism. SAR11 bacteria are notable for their specialized metabolism for oxidizing C1 and methylated compounds including DMSP for energy production (Sun et al. 2011). We demonstrate dynamic variations of phosphorylation sites in both DmdA and DmdC, key enzymes in the DMSP demethylation pathway, leading to production of energy and methanethiol, a precursor used in the atypical sulfur metabolism of these cells to supply sulfur for biosynthesis (Sun et al. 2016). Differentially regulated phosphorylation sites in the cleavage pathway were found only in YhdH, but not in DddK catalyzing the first step of DMSP cleavage pathway (Fig. 8). Both pathways of DMSP degradation were shown to be constitutively and simultaneously expressed in SAR11 bacteria (Sun et al. 2016). These observations support the possibility that phosphorylation is a regulatory modulator of SAR11 DMSP metabolism, which previously has been shown to be kinetically regulated (Bürgmann et al. 2007; Sun et al. 2016). Notably, MetK contained differentially regulated pS, pT and pY at positions of 64, 47 and 130, respectively (Fig. 8), suggesting that the S-adenosyl-l-methionine (SAM) production is also regulated by phosphorylation. Several SAM riboswitches involved in the post-transcriptional regulation of methyl metabolism and methionine biosynthesis have been described in SAR11 bacteria (Noell et al. 2023a). Thus, MetK phosphorylation and SAM riboswitches are implicated in post-transcriptional control of this pathway, which, like the citrate cycle, has both energy producing and anaplerotic functions. Overall, phosphorylation sites mapped to key enzymes in SAR11 carbon metabolism (Fig. 8), suggesting potential roles of protein phosphorylation in controlling metabolic flux. Functional validation of these sites will clarify whether phosphorylating enzymes controls metabolism in fluctuating environments, which is critical for interpreting future studies that aim to understand the fluxes of carbon and other compounds mediated by these cells.

### Streamlined oligotrophs and phosphorylation paradox

The evolutionary reduction of transcriptional regulation in marine oligotrophs such as SAR11 bacteria has been documented. Selection in these cells has favored retention of post-translational systems over transcriptional regulators (Giovannoni 2017; Noell et al. 2023a). Protein phosphorylation imposes demands for phosphorus, which is often a limiting nutrient in the oligotrophic oceans where SAR11 bacteria are most successful. Despite niche partition among diverse SAR11 ecotypes, clade Ia.1 represented by HTCC1062 still seasonally encounters phosphate depletion on productive coast (Bolaños et al. 2022). Thus, finding the largest bacterial phosphoproteome yet described in HTCC1062 was unexpected. The added cost of phosphorylating proteins to control metabolism may be offset by the benefits of rapidly responding to environmental stimuli and saving energy and material costs on coding and production of alternative transcriptional regulatory machinery. To compensate the environmental scarcity of phosphate, SAR11 bacteria have evolved a high-affinity phosphate transport system and capability for organic phosphorus utilization (Carini et al. 2014), and lipid remodeling to allocate phosphorus during phosphorus-starved periods (Carini et al. 2015). Moreover, SAR11 bacteria are the major consumer and producer of phosphonate in the surface ocean (Acker et al. 2022). Collectively, these studies suggest that SAR11 bacteria devote substantial resources to the acquisition and control of phosphorus, which support protein phosphorylation in addition to other phosphorus-dependent metabolic processes.

We hypothesize that protein phosphorylation may play a wide role in streamlined cells. It has been proposed that the reduction of transcriptional regulation in streamlined cells is driven by the diminished value of regulation to non-motile cells living in patchy environments, where transcriptional regulation is too slow to respond to short-term fluctuations in nutrient fields, or perhaps nutrient concentrations are too low for sensors to function properly (Noell et al. 2023a). The evolutionary trend to less transcriptional regulation may shift cellular priorities to reliance on regulation using structural RNAs and PTMs. Of these cellular mechanisms, PTMs offer the broadest control. In HTCC1062, more than 40% proteins were phosphorylated, suggesting global regulatory functions. Coupling of phosphorylation with other regulation, such as riboswitches, and two-component systems, is suggested by some of our observations. We speculate that overlapping systems of control for anaplerotic functions, where energy metabolism competes with anabolic reactions for intermediates, may be important for cells to achieve homeostasis in fluctuating environments.

## Conclusions

Our observations demonstrate very high levels of protein phosphorylation in a SAR11 proteome, patterns in the distribution of phosphorylation sites among proteins, and strong dynamics in protein phosphorylation driven by environmental variables. Nearly half of the proteins in these cells were detected in phosphorylated forms. These observations strongly support the prediction that SAR11 bacteria use phosphorylation to post-transcriptionally control metabolic processes. This model is in accord with other ideas about SAR11 metabolic regulation, which emphasize the apparently greater reliance of these cells on continuously expressed proteins and post-transcriptional regulation. The high levels of protein phosphorylation we observed in these very small, streamlined cells may in part be due to their reduced genomes, from which most auxiliary functions have been eliminated by selection, leaving a large core proteome. It could also reflect the usefulness of post-translational regulation in cells that may often be growth-limited and encounter ephemeral resources that leave them insufficient time to respond transcriptionally.

Future efforts should be devoted to identifying substrates of kinase and phosphatase systems, integrating phosphoproteomics with metabolomics to resolve metabolic network modulated by phosphorylation, and elucidating cross-talk between phosphorylation and other regulatory controls. Whether our findings are an indication of features broadly shared among diverse SAR11 ecotypes or even among streamlined oligotrophs needs to be examined. The broader significance of these efforts lies in their support a regulation model that is different from that of rapidly growing cell types, and broadens our perspective on how cells use regulation to optimize their performance in the face of environmental fluctuations, especially for the many newly discovered microbial taxa with highly reduced genomes that have been difficulty to cultivate.

## Supplementary Information

Below is the link to the electronic supplementary material.

Supplementary file 1 (PDF 2086 KB)

Supplementary file 2 (XLSX 648 KB)

## Data Availability

All mass spectra data for both proteomics and phosphoproteomics in this study have been deposited to the iProx database with accession number IPX0009465000 (https://www.iprox.cn) as well as the ProteomeXchange database with accession number PXD054839 (https://proteomecentral.proteomexchange.org).
