# Supplementary material for: Phosphoproteomics reveals essential regulatory roles of phosphorylation in marine oligotrophic bacteria
Source: Mar Life Sci Technol. 2025 Jul 15;8(2):628–41. doi: 10.1007/s42995-025-00305-w (PMC13198564; doi:10.1007/s42995-025-00305-w)
Supplement: Supplementary file 1 — (PDF 2086 KB) [file 42995_2025_305_MOESM1_ESM.pdf]

Supplementary Information

Supplementary figure

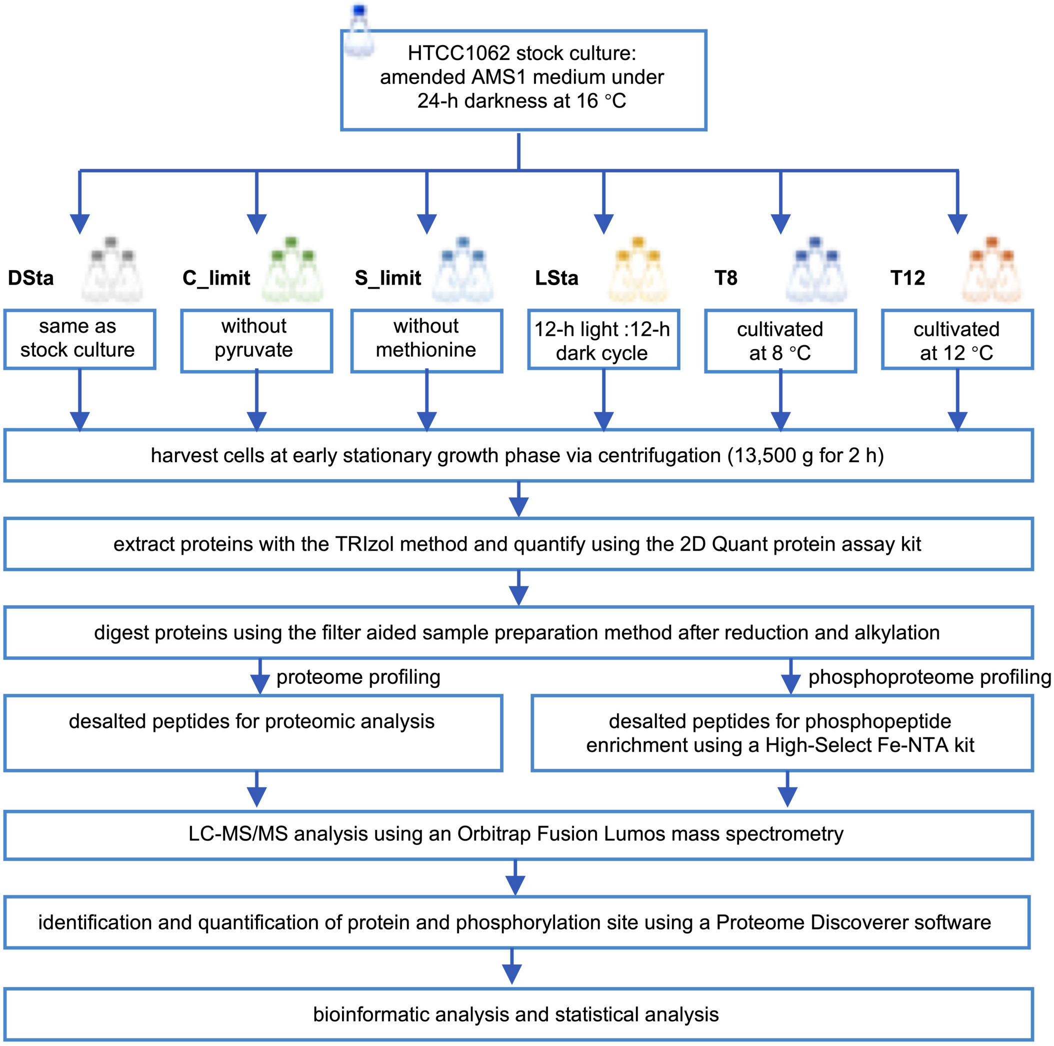

Fig. S1. Experimental design and work flow for proteomic and phosphoproteomic analyses of HTCC1062. Growth conditions for control group (DSta) are the same as stock cultures. In other treatments, growth conditions remain consistent with stock culture unless otherwise specified. For example, pyruvate and methionine are not added in media of treatments for carbon limitation (C-limit) and sulfur limitation (S_limit), respectively. A 12-h light:12-h dark cycle instead of 24-h darkness is used in the light treatment (LSta). Two temperature levels of 8 and 12 °C are used in treatments of T8 and T12, respectively. In each growth condition, cells are harvested at early stationary phase, and the same extracted protein material is used for both proteome and phosphoproteome profiling.

Legends of supplementary tables

Supplementary Table 1 (excel file). List of renewed sequences contained in the custom protein database for MS search.

Supplementary Table 2 (excel file). List of HTCC1062 proteins commonly identified in all the six growth conditions.

Supplementary Table 3 (excel file). List of identified phosphopeptides in this study.

Supplementary Table 4 (excel file). List of class I phosphorylation sites identified in HTCC1062.

Supplementary Table 5 (excel file). List of phosphorylation sites with quantitative information.

Supplementary Table 6 (excel file). Comparison of HTCC1062 phosphoproteome with other bacterial phosphoproteomes.

Supplementary Table 7 (excel file). Statistics of S/T/Y content and percentage of pS/pT/pY for each phosphoprotein.

Supplementary Table 8 (excel file). The distribution of phosphoproteins among KEGG pathways.

Supplementary Table 9 (excel file). Differential phosphorylation sites of HTCC1062.
